# Hypertrophy of the ligamentum flavum in lumbar spinal canal stenosis is associated with abnormal accumulation of specific lipids

**DOI:** 10.1038/s41598-021-02818-7

**Published:** 2021-12-06

**Authors:** Tomohiro Yamada, Makoto Horikawa, Tomohito Sato, Tomoaki Kahyo, Yusuke Takanashi, Hiroki Ushirozako, Kenta Kurosu, Md. Al Mamun, Yuki Mihara, Shin Oe, Hideyuki Arima, Tomohiro Banno, Go Yosida, Tomohiko Hasegawa, Yu Yamato, Yukihiro Matsuyama, Mitsutoshi Setou

**Affiliations:** 1 Department of Orthopedic Surgery, Hamamatsu University School of Medicine, 1-20-1, Handayama, Higashi-ku, Hamamatsu, Shizuoka 431-3192 Japan; 2 Department of Cellular and Molecular Anatomy, Hamamatsu University School of Medicine, 1-20-1, Handayama, Higashi-ku, Hamamatsu-city, Shizuoka 431-3192 Japan; 3 International Mass Imaging Center, Hamamatsu University School of Medicine, 1-20-1 Handayama, Higashi-ku, Hamamatsu, Shizuoka 431-3192 Japan; 4 Department of Molecular Biotechnology, Graduate School of Advanced Science of Matter, Hiroshima University, 1-4-4, Kagamiyama, Higashi-Hiroshima, Hiroshima 739-7763 Japan; 5 Department of Systems Molecular Anatomy, Institute for Medical Photonics Research, Preeminent Medical Photonics Education and Research Center, 1-20-1 Handayama, Higashi-ku, Hamamatsu, Shizuoka 431-3192 Japan

**Keywords:** Diseases, Pathogenesis

## Abstract

Ligamentum flavum hypertrophy (HLF) is the most important component of lumbar spinal canal stenosis (LSCS). Analysis of hypertrophied ligamentum flavum (HLF) samples from patients with LSCS can be an important que. The current study analyzed the surgical samples of HLF samples in patients with LCSC using quantitative and qualitative high performance-liquid chromatography and mass spectrometry. We collected ligamentum flavum (LF) tissue from twelve patients with LSCS and from four patients with lumbar disk herniation (LDH). We defined LF from LSCS patients as HLF and that from LDH patients as non-hypertrophied ligamentum flavum (NHLF). Total lipids were extracted from the LF samples and evaluated for quantity and quality using liquid chromatography and mass spectrometry. The total lipid amount of the HLF group was 3.6 times higher than that of the NHLF group. Phosphatidylcholines (PCs), ceramides (Cers), O-acyl-ω-hydroxy fatty acids (OAHFAs), and triglycerides (TGs) in the HLF group were more than 32 times higher than those of the NHLF group. PC(26:0)+H+, PC(25:0)+H+, and PC(23:0)+H+ increased in all patients in the HLF group compared to the NHLF group. The thickness of the LF correlated significantly with PC(26:0)+H+ in HLF. We identified the enriched specific PCs, Cers, OAHFAs, and TGs in HLF.

## Introduction

Lumbar spinal canal stenosis (LSCS) is a common cause of low back and lower extremity pain, particularly in elderly patients^1^. LSCS occurs as a result of degenerative changes in the lumbar spine, including bulging of the intervertebral disks, bony degeneration of the facet joints, and hypertrophied ligamentum flavum (HLF)^2–4^. HLF is considered a major contributor to the development of LSCS^5^. Previous studies have indicated that HLF shows elastic fiber loss and increased collagen content^6,7^. Several factors such as aging^8^, mechanical stress^2,9^, transforming growth factor-beta^10^, and matrix metalloproteinases^11^ are considered to be involved in LF degeneration.

Lipid molecules are involved in many cellular functions including regulation of the physical properties of the cellular membrane, cell proliferation, migration, differentiation, inflammation, and interaction^12–15^. Laminectomy has been mainly a surgical treatment aiming for removing HLF affecting neural compression, and removing accumulating lipids in HFL would be leading to an alternative treatment. However, clinical significance of evaluating the details of lipids profiles accumulating in human HLF remains to be unknown. Clarify the specific lipids accumulating in HLF might yield an intervention for LSCS other than the surgical technique. Therefore, this study aimed to analyze and quantify the lipids accumulating in HLF using lipidomics technologies. Here we show an increase in total lipid accumulation and in the levels of specific phosphatidylcholines (PCs), ceramides (Cers), O-acyl-ω-hydroxy fatty acids (OAHFAs), and triglyceride (TGs) in HLF.

## Results

### Patient background and clinical data

**Figure. 1:**
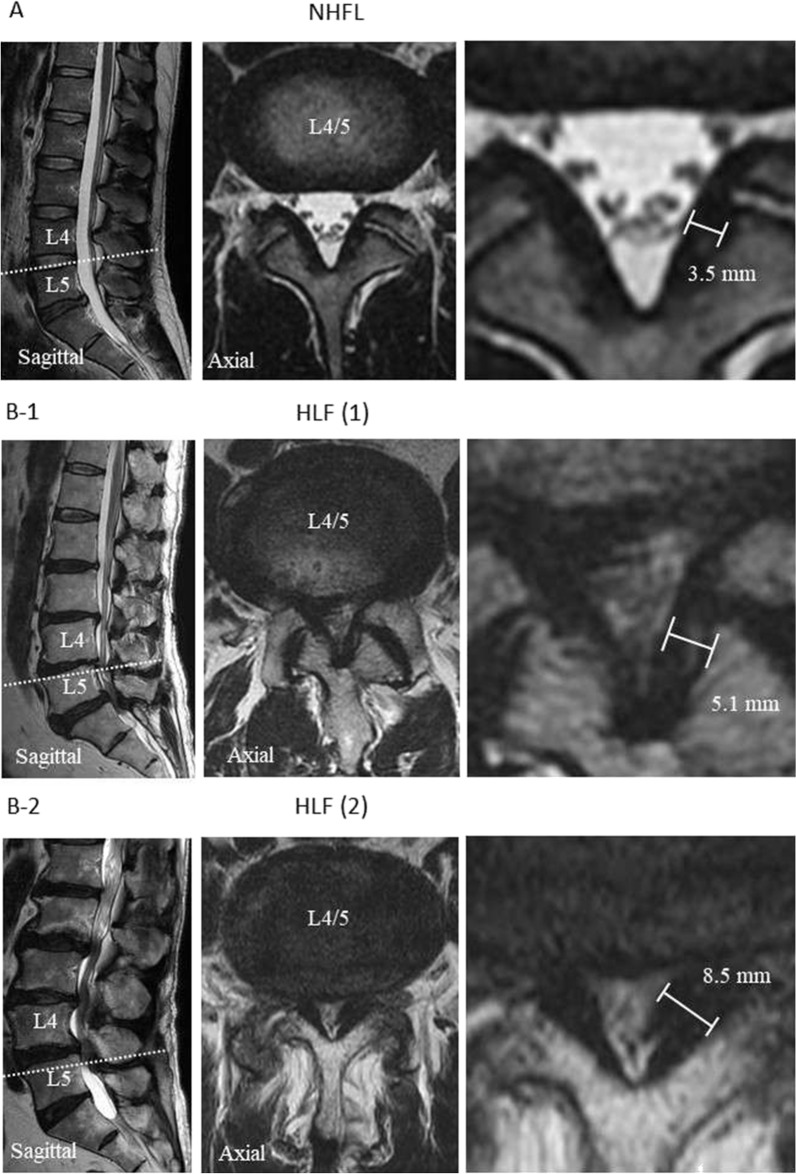
Magnetic resonance imaging (MRI) showing sagittal and axial views of lumbar spinal canal. The axial views illustrate the L4/5 facet joints (see white dotted line). A: T2-weight MRI showing non-hypertrophied ligamentum flavum (NHLF) of non-lumbar spinal canal stenosis (LSCS) B-1 and B-2: T2-weight MRI showing hypertrophied ligamentum flavum (HLF) of LSCS. The white scale bars indicate the thickness of the LF.

Preoperative patient background information, laboratory data, and the thickness of LF are shown in Table 1 for both the HLF and NHLF groups. Analysis of MRI images revealed that the thickness of LF in the HLF group was two-times higher than that in the NHLF group (*P* < 0.01; Fig. 1). There was an increased tendency and the serum triglyceride (TG) concentration was more than two-times greater in the HLF group than in the NHLF group (*P* < 0.01) (Table 1).

**Table 1:** Background and laboratory data of both groups.

		HLF	NHLF	*P* value†
Background	Patients (male)	12 (6)	4 (2)	0.98
	Age (years)	73.3 ± 6.1	39.3 ± 0.5	**< 0.001**
	Height (cm)	158 ± 11.2	162 ± 15.3	0.67
	Weight (kg)	59.5 ± 12.2	65.0 ± 8.4	0.60
	BMI	23.2 ± 3.1	25.1 ± 6.1	0.55
	Complication of DM	3	0	0.27
	LF thickness (mm)	6.1 ± 2.3	3.3 ± 0.5	**0.01**
BC	WBC (/μL)	7078 ± 2291	7857 ± 2970	0.65
	Hgb (g/dL)	14.2 ± 1.8	14.0 ± 1.3	0.88
	Hct (%)	43.0 ± 4.8	41.6 ± 3.2	0.66
	Plt (×10^4^/μL)	21.6 ± 4.8	28.3 ± 11.4	0.20
Chemistry	TP (g/dL)	6.8 ± 1.0	7.1 ± 0.2	0.56
	ALB (g/dL)	4.2 ± 0.7	4.3 ± 0.3	0.9
	T-Bil (g/dL)	0.7 ± 0.3	0.7 ± 0.3	0.95
	AST (U/L)	24.6 ± 9.5	16.7 ± 1.5	0.19
	ALT (U/L)	21.4 ± 8.9	19.7 ± 3.2	0.63
	γ-GTP (U/L)	26.6 ± 15.3	21.7 ± 12.7	0.63
	ALP (U/L)	248.7 ± 8.9	157 ± 11.3	0.23
	BUN (g/dL)	18.9 ± 6.2	9.2 ± 1.4	**0.03**
	Cre (g/dL)	0.8 ± 0.3	0.6 ± 0.1	0.29
	T-chol (mg/dL)	198 ± 37.1	199 ± 21.5	0.96
	TG (mg/dL)	240 ± 90.8	99 ± 12	**0.01**

Data are presented as the mean ± SD.

HLF: hypertrophied ligamentum flavum, NHFL: non hypertrophied ligamentum flavum. BMI: body mass index, DM: diabetes mellitus, LF: ligamentum flavum, BC: blood count, WBC: white blood cell, Hgb: hemoglobin, Hct: hematocrit, Plt: platelet, TP: total protein, ALB: albumin, T-Bil: total bilirubin, AST: aspartate aminotransferase, ALT: alanine aminotransferase, GTP: glutamic pyruvic transaminase, ALP: alkaline phosphatase, BUN: blood urea nitrogen, Cre: creatinine, T-chol: total cholesterol, TG: triglyceride.

*Bold type indicates statistical significance. †Comparison between two groups.

### LC–MS/MS measurement of lipid composition in the HLF and NHLF groups

**Figure. 2:**
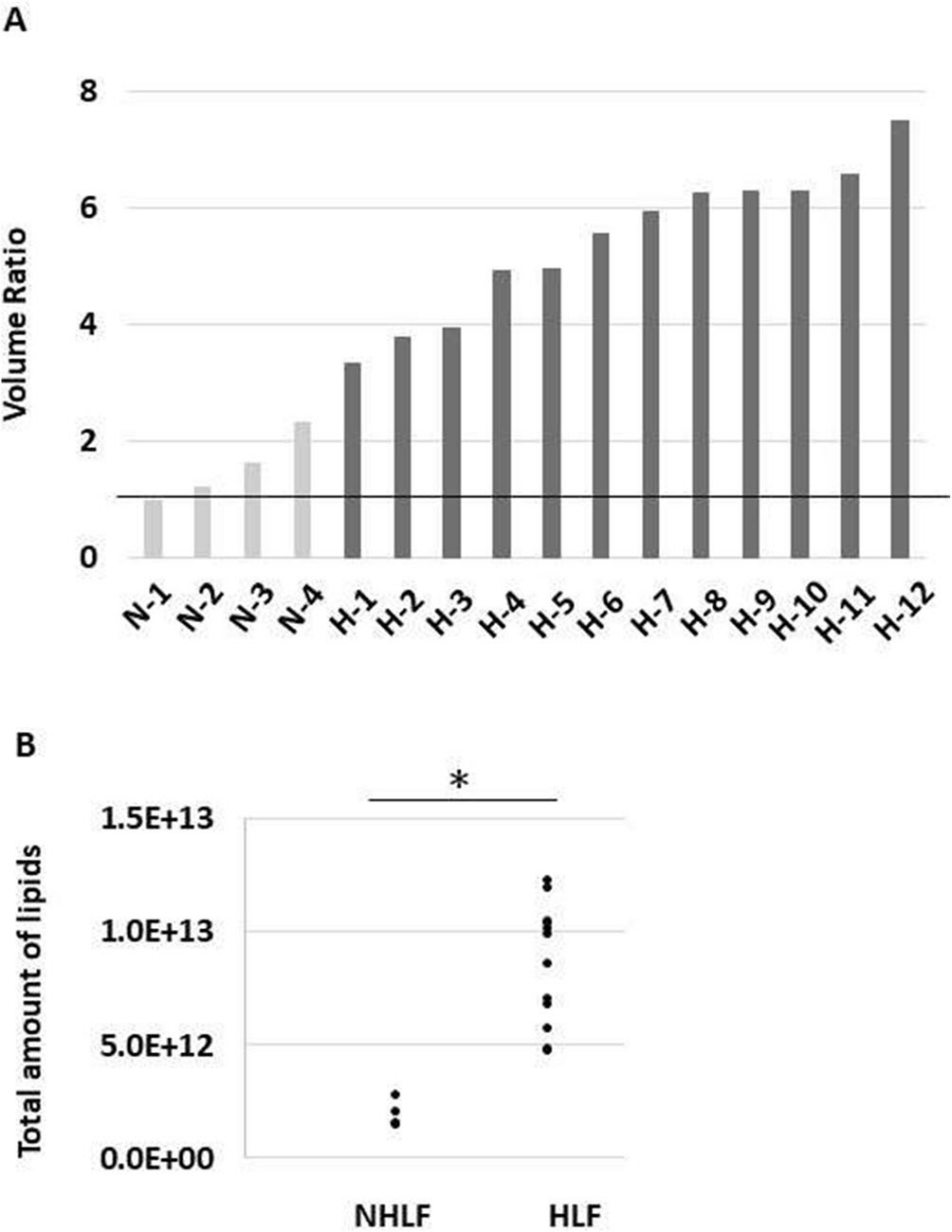
Total amount of lipids and ligament volume ratio between the non-hypertrophied ligamentum flavum (NHLF) and hypertrophied ligamentum flavum (HLF) groups. (**A**) Ligament volume ratio in each sample. (**B**) Total amount of lipids between the NHLF and HLF groups after correcting for volume ratio. *P < 0.01. N: each sample from NHLF group; H: each sample from HLF group.

We surgically obtained LF samples from patients and subjected them to lipid extraction (Supplemental Fig. 1). Extracted lipids were measured using LC–MS/MS and the spectra were analyzed using LipidSearch software. Supplemental Fig. 2 shows the chromatograms of samples from HLF and NHLF groups. The quantities of lipids were normalized to the IS, PC (12:0/12:0), and the averages of each lipid were calculated. After each lipid quantity was adjusted by the ligament volume ratio, we evaluated the quantitative differences in the lipids commonly observed in both groups. Each ligament volume ratio in HLF increased compared with that in the NHLF group (Fig. 2A). Upon correcting for the ligament volume ratio, the total amount of lipids in each sample of HLF group was significantly increased compared with that in the NHLF group (Fig. 2B).

**Figure. 3:**
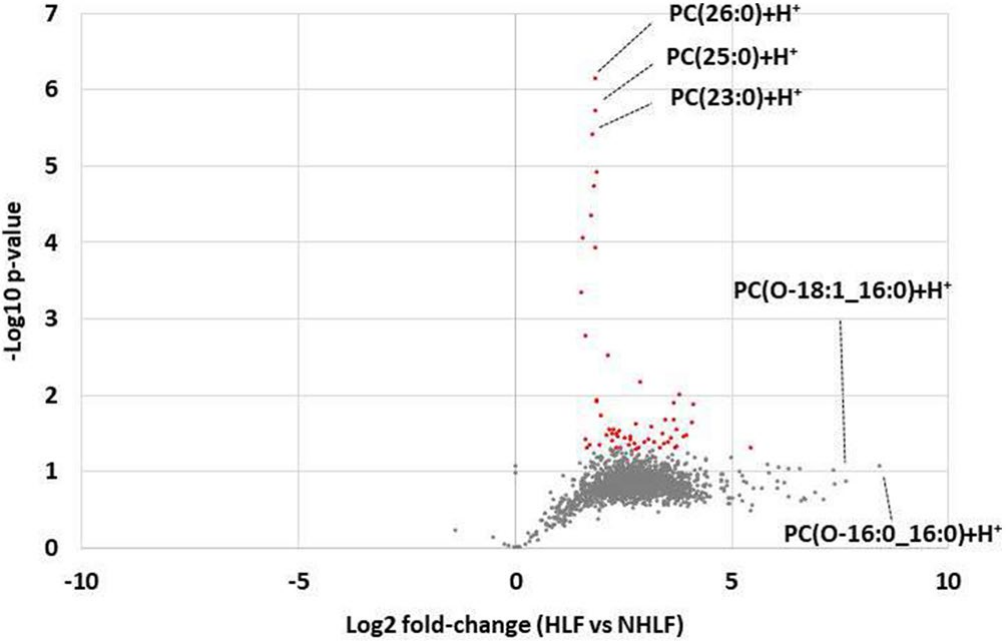
Volcano plot representing the changes in lipids between the hypertrophied ligamentum flavum (HLF) and non-hypertrophied ligamentum flavum (NHLF) groups. Red dot presents lipids in HLF increased significantly two times compared to NHLF (*P* < 0.01).

**Figure. 4:**
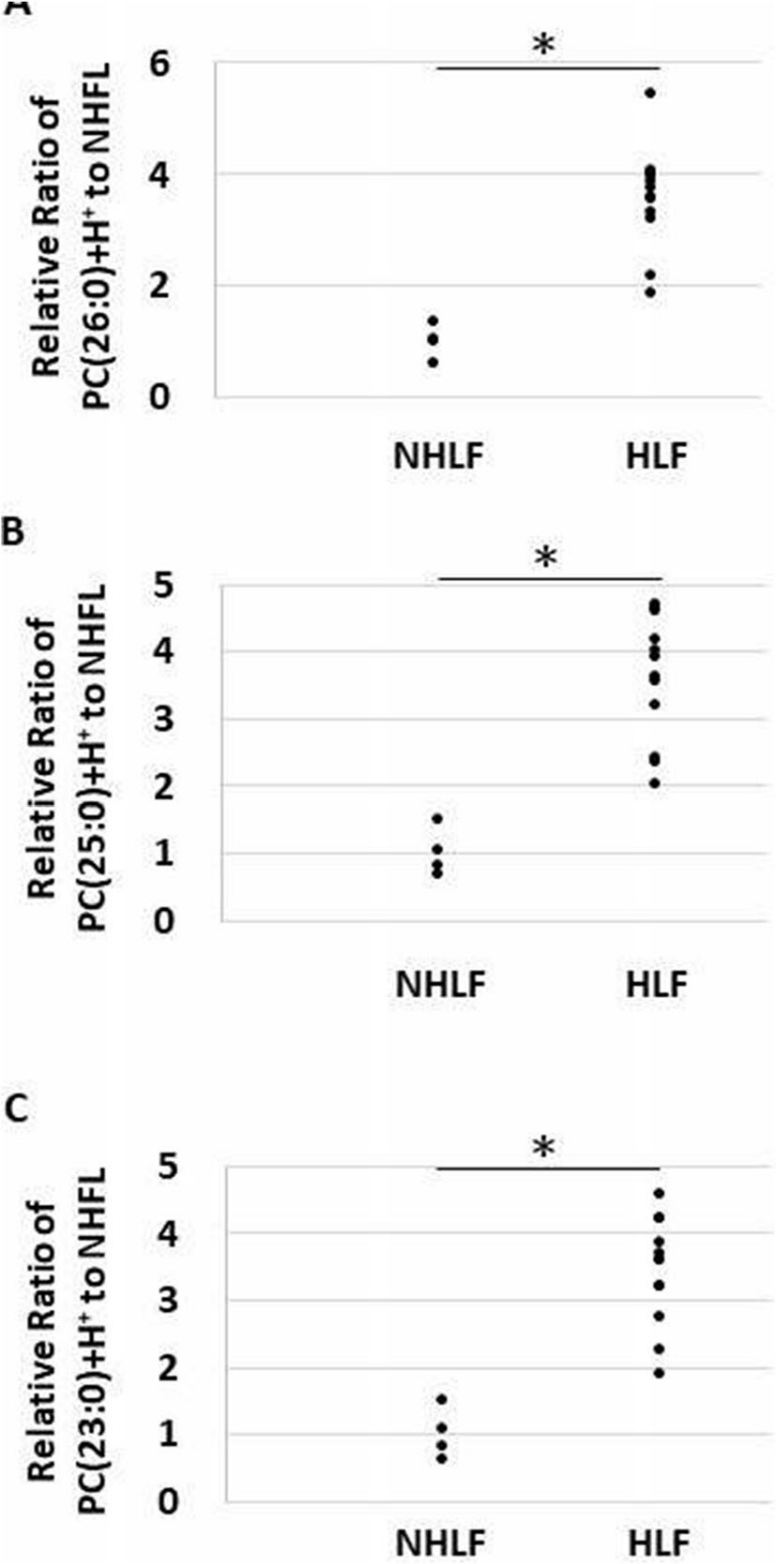
Dot plot showing the ratio of each lipid intensity to the average value of non-hypertrophied ligamentum flavum (NHLF) group. (**A**) PC(26:0)+H+, (**B**) PC(25:0)+H+, (**C**) PC(23:0)+H+. *P < 0.001.

**Figure. 5:**
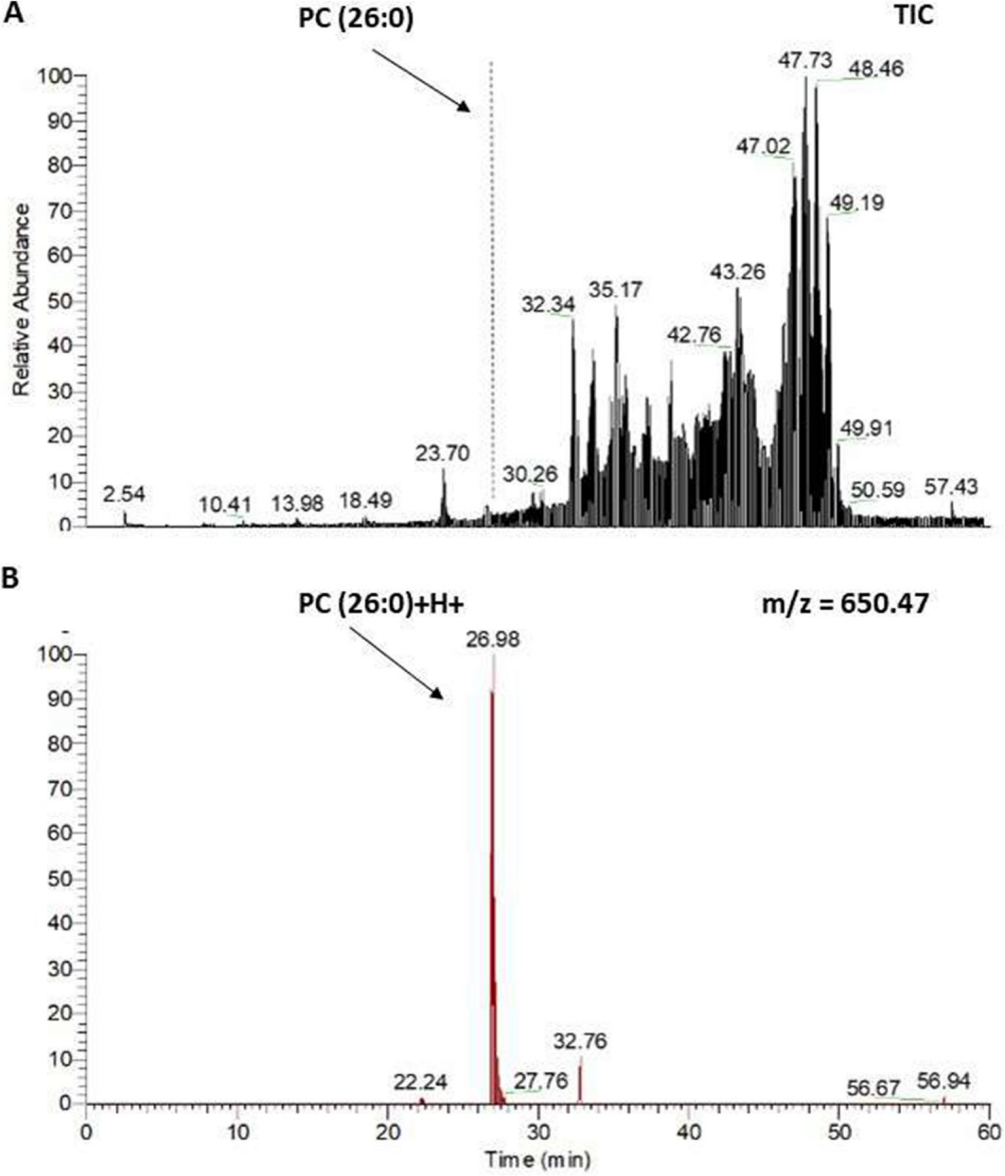
Detection of PC(26:0) in hypertrophied ligamentum flavum (HLF) by liquid chromatography (LC) analysis. LC analysis of HLF using the M+H+ ion at m/z 650.47. (**A**) Base peak chromatogram of PC (26:0) isolated from HLF with internal standard added. (**B**) Reconstructed chromatogram for m/z 650.4775 showing PC (26:0)+H+.

Quantitative analysis using LipidSearch revealed that there were 1304 common lipids species and that 58 were increased significantly in the HLF group compared to that in the NHLF group (Fig. 3). In the HLF group, PC(O-16:0_16:0)+H+ was more than 300 times higher, and PC(O-18:1_16:0)+H+ was more than 160 times higher compared to those in the NHLF group. Supplemental Table 1 shows the lipids that were more than 32 times increased in the HLF compared to that in the NHLF group. Several PCs, (O-acyl)-ω-hydroxy fatty acids (OAHFAs), and TGs showed a tendency to increase in the HLF group. In all subjects in the HLF group, PC(26:0)+H+, PC(25:0)+H+, and PC(23:0)+H+ were higher than the corresponding levels measured in the NHLF group (*P* < 0.001) (Fig. 4).The peaks of ions at m/z 650.4755 were recognized at retention time of 26.98 min corresponding to PC(26:0)+H+ (Fig. 5). Combination of more than two those lipids including total PCs, OAHFAs, and TGs was likely to increase in HLF group compared to those of NHLF group (Supplemental Fig. 3).

### MALDI-IMS analysis for the HLF and NHLF groups

**Figure. 6:**
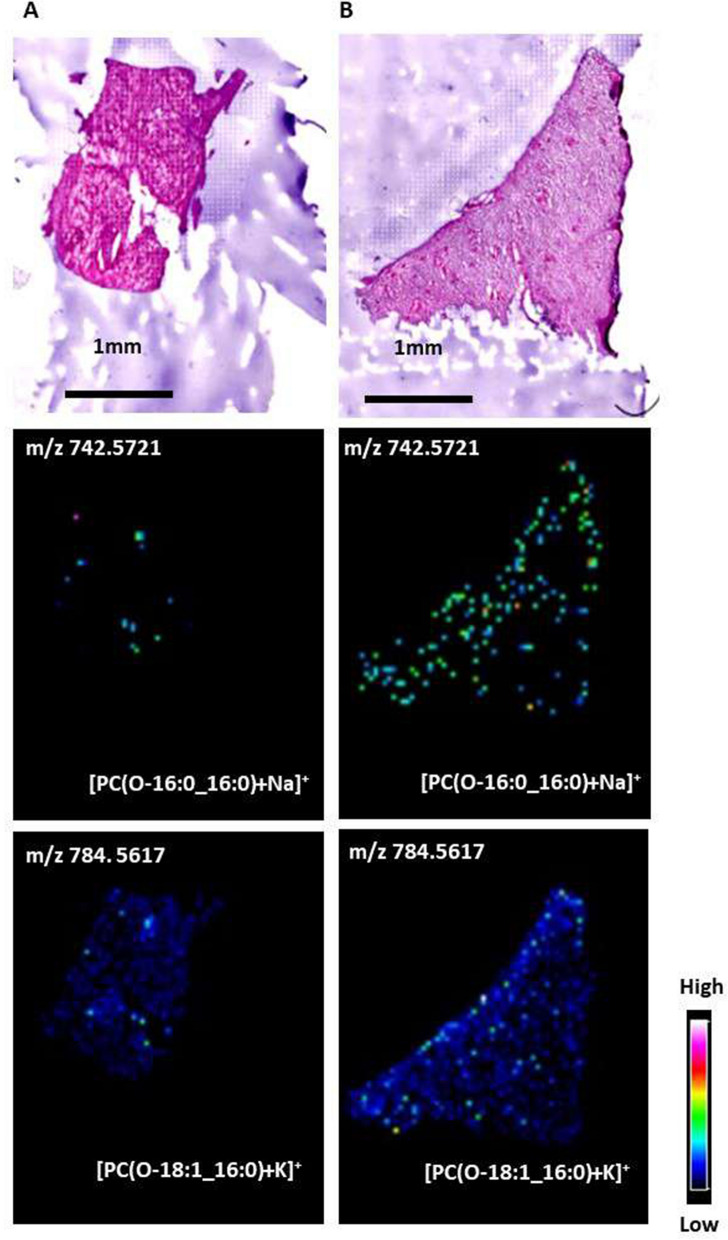
Characterization of PC(O-16:0_16:0) and PC(O-18:1_16:0) in the non-hypertrophied ligamentum flavum (NHLF). (**A**) and hypertrophied ligamentum flavum (HLF) (**B**) groups. An optical image of a hematoxylin and eosin-stained LF section and ion images of PC(O-16:0_16:0)+Na+ and PC(O-18:1_16:0)+K+ obtained from consecutive section by matrix-assisted laser desorption ionization imaging mass spectrometry (MALDI-IMS) are depicted. In the LF of HLF group, the signal intensity of PC(O-16:0_16:0)+Na+ (m/z 742.5721) and PC(O-18:1_16:0)+K+ (m/z 784.5617) increased further than those of NHLF group. The detected ions were of natrium and potassium adducts.

We analyzed the lipid distribution in the representative cases of HLF and NHLF groups using MALDI-IMS. Figure 6 shows the signal intensity and distributions of the representative detected phospholipids. In MALDI-IMS using DHB as a matrix, PCs are preferentially detected as alkali metal adduct ions, such as sodium and potassium, rather than hydrogen^16^. Therefore, we observed PC(O-16:0_16:0) and PC(O-18:1_16:0) as the alkali metal adductions. In the HLF group, PC(O-16:0_16:0) and PC(O-18:1_16:0) increased compared to those in the HNLF group.

### Correlation among thickness of LF, serum TG, PC(26:0)+H+, and the prevalence of type 2 diabetes mellitus (T2DM)

**Figure. 7:**
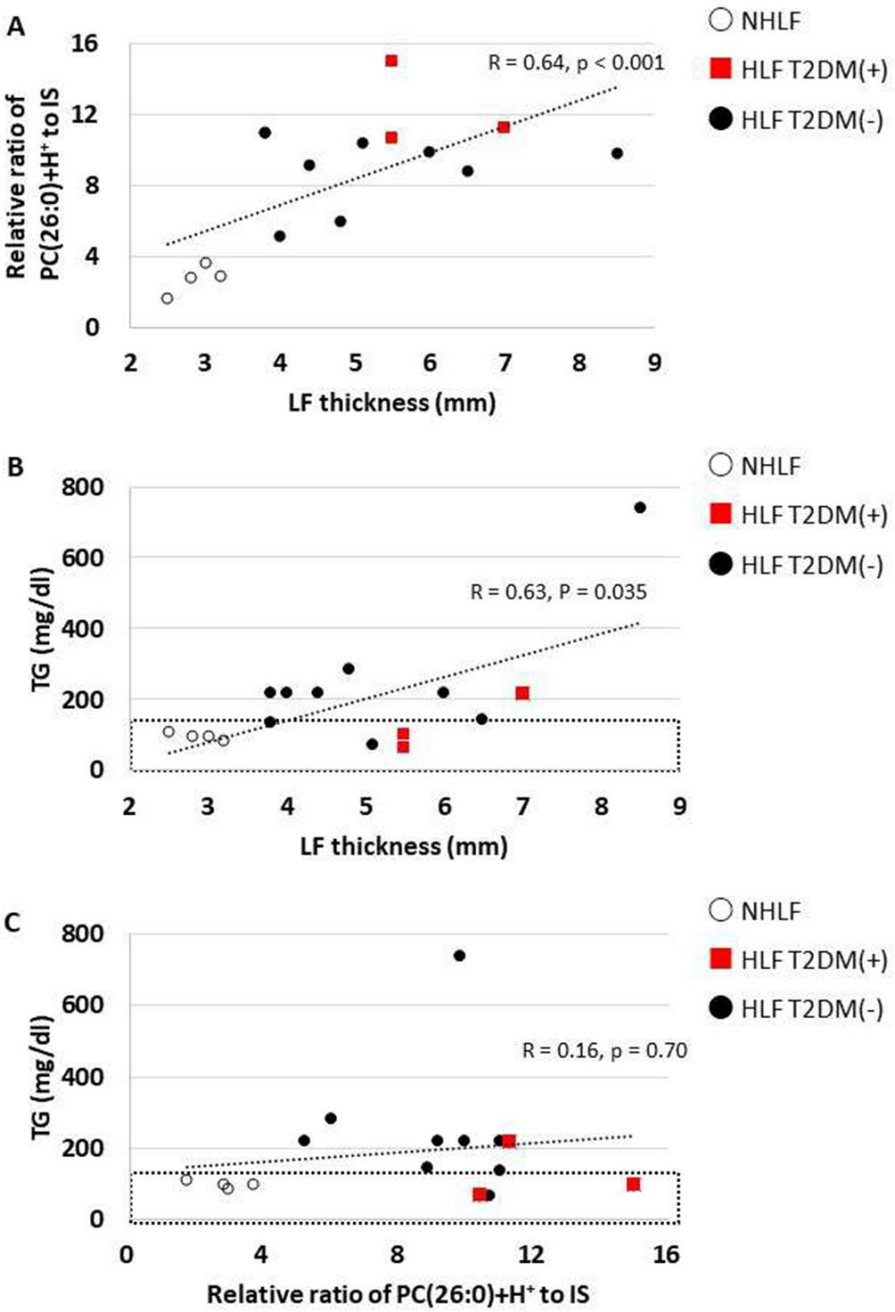
Dot plots of data from patients with non-hypertrophied ligamentum flavum (NHLF) or hypertrophied ligamentum flavum (HLF) with type 2 diabetes mellitus (T2DM +), and HLF without T2DM( −). (**A**) Correlation between thickness of ligamentum flavum (LF) and the relative ratio of PC(26:0)+H+ to the internal standard (IS). (**B**) Correlation between thickness of LF and the concentration of serum triglyceride (TG) concentration. The black dotted rectangle represents the normal range of serum TG concentrations. (**C**) Correlation between the relative ratio of PC(26:0)+H+ to the IS and serum TG concentration. The black dotted rectangle represents the normal range of serum TG concentration.

We evaluated the correlations among LF thickness, the concentration of serum TG, and area ratio of PC(26:0)+H+ to the IS in HLF group (Fig. 7). The thickness of LF was significantly correlated with PC(26:0)+H+ levels (r = 0.64, *P* < 0.001), and it was significantly higher in patients with T2DM than in those without T2DM (*P* = 0.035) (Fig. 7A). The LF thickness was also significantly correlated with the concentration of serum TG (r = 0.63, *P* = 0.015) in the HLF group (Fig. 7B). PC(26:0)+H+ levels were not correlated with the concentration of serum TG (Fig. 7C). Furthermore, patients with T2DM did not show higher concentrations of serum TG than the subjects without T2DM (Fig. 7B,C).

### The distribution of levels of PC(O-16:0_16:0)+H+ and PC(O-18:1_16:0)+H+ in the HLF and NHLF group

**Figure. 8:**
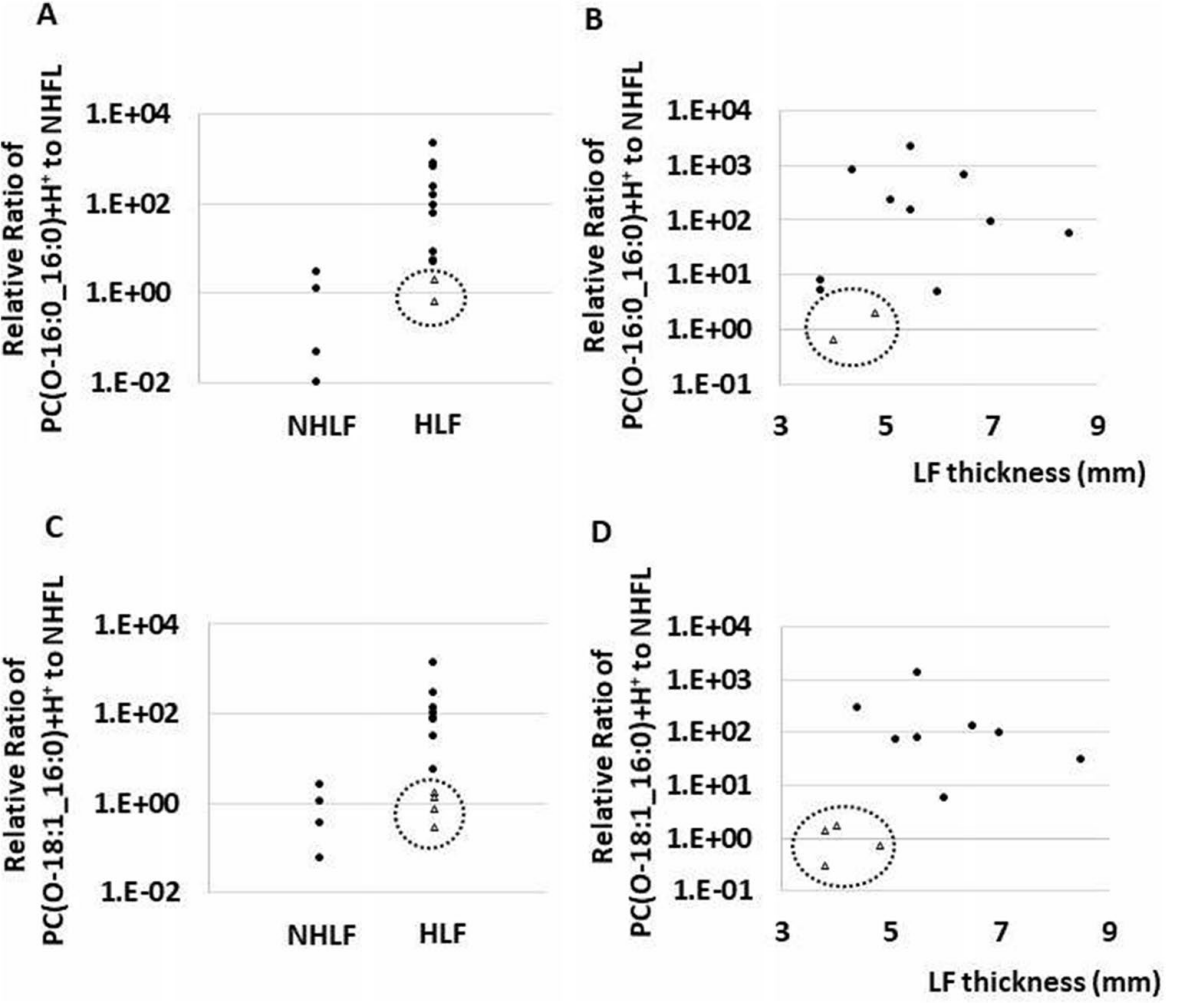
Dot plot showing the ratio of each lipid intensity to the average value of control, and the relation of symptom duration to surgery (months). (**A**, **B**) In case of PC(O-16:0_16:0)+H+, two cases in the hypertrophied ligamentum flavum (HLF) group presented a low ratio (enclosed by a dotted circle), and low ratio cases showed relatively thin ligamentum flavum (LF). (**C**, **D**) In case of PC(O-18:1_16:0)+H+, four cases in HLF group presented a low ratio (enclosed by a dotted circle), and low ratio cases showed relatively thin LF.

PC(O-16:0_16:0)+H+ and PC(O-18:1_16:0)+H+ were highly variable in the HLF group. Some cases showed low levels of both PC(O-16:0_16:0)+H+ and PC(O-18:1_16:0)+H+, and those cases showed relatively thin LF in HLF group (Fig. 8).

## Discussion

In the current study, we found that hypertrophy of LF was associated with quantitative and qualitative lipid changes and demonstrated total lipid accumulation in the HLF group. Specific PCs, such as PC(O-16:0_16:0)+H+ and PC(O-18:1_16:0)+H+, were remarkably increased in the HLF group, evident from the MALDI-IMS analysis. Of note, the expression of PC(O-16:0_16:0)+H+ and PC(O-18:1_16:0)+H+ in HLF was highly uniform; MALDI-IMS proved their accumulation, visually. We also demonstrated that PC(26:0)+H+, PC(25:0)+H+, and PC(23:0)+H+ showed a reproducible tendency to be elevated in the HLF group.

The distribution of the levels of PC(O-16:0_16:0)+H+ and PC(O-18:1_16:0)+H+ were highly variable in the HLF group. However, some cases showed low levels of both PC(O-16:0_16:0)+H+ and PC(O-18:1_16:0)+H+ (Fig. 8). Interestingly, the subjects with low PC(O-16:0_16:0)+H+ and PC(O-18:1_16:0)+H+ levels were less likely to show LF thickness compared with the other subjects. A possible explanation for this fact might be that PC(O-16:0_16:0)+H+ and PC(O-18:1_16:0)+H+ increase proportionally to LF thickness.

PC(26:0)+H+ was the main phospholipid found to be increased in the HLF group and correlated with LF thickness. Several risk factors such as T2DM, hypertension, obesity, and smoking habits have been identified as contributors to the pathogenesis of LSCS^17–20^. T2DM played an essential role in the induction of HLF in several east and west Asian populations^20^. Previous research has reported that T2DM induces adipose tissue signaling and increases the rate of de novo PC biosynthesis^21^. Similarly, we demonstrated that in the HLF group, the median values of the area ratio of saturated PC(26:0)+H+ in patients with T2DM tended to increase in comparison to those without T2DM (11.2 vs. 8.8, respectively) (Fig. 7A). These results suggest that increased PCs may not only correlate with T2DM, but it may also be a novel risk factor for LSCS. PCs is the most abundant phospholipid in human serum, and saturated fatty acids act mainly on stored fat that is used for energy^22^. In our study, we showed that several saturated PCs significantly increased in HLF compared to NHLF (Fig. 3). We also found that specific saturated PC, PC(26:0)+H+ was significantly accumulated in HLFs and correlated with HLF thickness (Fig. 7A). Previous studies have shown that PC(26:0) is increased in mice fed a high-fat diet^23^. Thus, patients with HLF may have higher levels of stored fat, and removal of stored fat may decrease saturated PCs and prevent LF becoming proliferated, or may decrease the volume of the LF.

Zhou et al. described that Lysophosphatidic acid (LPA) concentration in the cerebrospinal fluid of patients with HLF was higher than those in subjects with NHLF, and LPA induced hypertrophy of LF via LPA receptor1 to Akt signal pathway^24^. However, their study did not show the lipid distributions in HLF. In that respect, our study shows the importance of evaluating the lipids profiles of HLF. In this study, there were several reasons that LPAs were not identified. First, the modified Bligh–Dyer method mainly extracted neutral lipids, suggesting that acid lipids like LPAs were not determined. Second, LPAs have been unstable and quickly break down to other lipids at room temperature. Then, harvested LF at room temperature may allow LPAs to turn down for other lipids.

The previous report described that lysophosphatidylcholines (LPCs), hydrolyzed from PCs via phospholipase C, increases growth factors contributing to thickness for LF. We also identified enriched LPC(17:0)+H, which may be possible for deriving from PC(17:0_18:1)+H (Supplemental Table 1). In HLF, we identified enriched OAHFAs (Supplemental Fig. 4) leading to age-related macular degeneration with yellow corneal rings^25^. Interestingly, HLF also appears as yellow in gross (Supplemental Fig. 1), originated from lipofuscin. Lipofuscin formed by oxidation of unsaturated fatty acid exerts autofluorescence, and the fluorescent particle generates fluorescent light of 500–650 nm wavelength. Therefore, it might be possible that yellow ligament appearance derived from OAHFAs.

We acknowledge that there are limitations to this study. First, we included LF from the LDH of younger patients in the NHLF group, leading to a significant difference in the mean patient age between the two groups. Wakabayashi investigated the difference of Lipid accumulation product (LAP) on aging between men and women^26^. In men, LAP was highest at middle age and declined thereafter, while tended to be higher with aging in women. However, older patients with LDH frequently present hypertrophied ligamentum flavum simultaneously in actual clinical practice. Therefore, we could not include older patients in the NHLF group and cannot deny the possibility that aging increased the PC levels. Second, the LF samples were left at room temperature during a surgical operation, and several lipids are unstable under such conditions. Therefore, their lipid compositions might have changed due to endogenous enzymes.

More research on this topic with larger sample sizes needs to be undertaken before the association between specific lipids and hypertrophied LF. However, our results are potentially significant in that specific PCs were increased compared to the other lipids in the HLF group and correlated with LF thickness.

In conclusions, PCs, Cers, OAHFAs, and TGs were increased in HLF. Those findings would provide an insight into LSCS pathogenesis that can be exploited to design an alternative treatment strategy in the future.

## Materials

All methods were carried out in accordance with relevant guidelines and regulations.

### Chemicals

Methanol, chloroform, glacial acetic acid, and ultrapure water were purchased from Wako Pure Chemical Industries (Osaka, Japan). Phosphatidylcholine (PC) PC(12:0/12:0), 1,2-dilauroyl-sn-glycero-3-phosphocholine (Avanti Polar Lipids, Alabaster, AL, USA), was used as a lipid calibration standard. 2,5-dihydroxybenzoic acid (DHB), a matrix-assisted laser desorption ionization matrix, was purchased from Bruker Daltonics (Fremont, CA, USA). All chemicals used in this study were of the highest purity available.

### Participants

The Research Ethics Committee at the Hamamatsu University School of Medicine reviewed and approved the study for human subjects (#17-065). Informed consent was duly signed after procuring institutional ethical clearance. All experimental protocols were approved by a named institutional. All operations were conducted at our institution. Preoperatively, we measured the thickness of LF tissue using magnetic resonance imaging (MRI). LF thickness, seen at low density with MRI T1 image, was measured at the facet level of the axial image (Fig. 1). One experienced spine surgeon measured the LF thickness. LF was collected from the L4/5 or L5/S1 of each patient. Non-hypertrophied LF (NHLF) were collected from patients with lumbar disc herniation (LDH) and HLF were collected from patients with LSCS. LDH was defined as radiculopathy due to disc herniation, and LSCS as cauda equina syndrome. We excluded patients with LSCS who had only imaging findings of LDH. NHLF was obtained from 4 patients (2 males and 2 females; average age: 39.3 ± 0.48 years; range: 30–43 years) with LDH who underwent laminectomy to prepare the posterior pathway for discectomy without fusion. HLF was obtained from 12 patients (6 males and 6 females; average age: 73.3 ± 6.1 years old; range: 63–83 years) who underwent decompression laminectomy for symptomatic degenerative LSCS.

### Clinical blood testing

Preoperatively, fasting venous blood samples were obtained for analysis of blood count and chemistry. All blood samples were analyzed by the clinical laboratory center of our hospital.

### Sample collection and lipid extraction

Each LF tissue sample obtained during surgery was stored in normal saline solution during the operation. Following surgery, the samples were washed with saline solution to remove blood, frozen with powdered dry ice, and stored at − 80℃ until lipid extraction. Total lipids were extracted using a modified Bligh–Dyer method^27,28^ LF tissue was processed using a surgical knife and 0.5 g of the tissue was transferred into a glass tube, to which was added 2.0 mL of methanol, 1.0 mL of chloroform, and 40 µmol phosphatidylcholine PC(12:0/12:0) as the internal standard (IS).

### Lipid analysis by liquid chromatography-tandem mass spectrometry (LC–MS/MS)

All extracted lipids were analyzed using LC–MS/MS. LC analysis was performed using a Dionex Ultimate 3000 instrument (Thermo Fisher Scientific, Waltham, MA, USA), and Q Exactive was used for MS/MS analysis (Thermo Fisher Scientific). We used the Q Exactive Orbitrap, which allows for data-dependent acquisition scans of MS1 followed by MS2 scans per cycle. Using this platform, the resolution of the Orbitrap mass analyzer was set to 70,000 for MS1 scans and 17,500 for MS2 scans. A chromatographic method was developed using a Thermo Scientific Acclaim column (2.1 × 150 mm i.d., 3 μm particles) and the components were subjected directly to MS/MS analysis. The samples (10 μL) were directly injected using the autosampler and the components were separated using a set gradient with mobile phase A (acetonitrile:methanol:water, 1:1:2 v/v/v, containing 0.1% formic acid and 5 mM ammonium acetate) and mobile phase B (acetonitrile:isopropanol, 1:9 v/v, containing 0.1% formic acid and 5 mM ammonium). The flow rate was 300 µL/min. The gradient used was 20–100% solvent B over 50 min. After washing with solvent B for 10 min, the column was re-equilibrated with 20% B for 10 min.

### Data analysis

The following software (Thermo Fisher Scientific) were used for data analysis: XCalibur 2.2 for automated data acquisition of LC–MS/MS data and analysis of mass spectra of MS1 and MS/MS scans, CompoundDiscoverer 2.1 for a brief comparison of LC–MS/MS data, and LipidSearch for identification and quantification of lipids. The LF volume ratios were calculated using LF area, as determined by MRI sagittal images, raised to the power of 1.5.

### Tissue preparation for matrix-assisted laser desorption ionization imaging mass spectrometry (MALDI-IMS)

Harvested LF was embedded in Super Cryoembedding Medium after dissection and were flash frozen on dry ice and stored at − 80 °C. Tissue sectsions (10 μm) were prepared at − 20 °C using a cryostat (CM1950; Leica, Wetzler, Germany) and placed alternately onto glass slides coated with indium-tin-oxide (ITO) (Matsunami) for MALDI-IMS assessment, and onto un-coated glass slides (Matsunami) for hematoxylin and eosin (HE) staining. For MALDI-IMS analysis, LF section of HLF and that of NHLF were mounted onto the same ITO glass slide.

### IMS sample preparation and MALDI-IMS analysis

DHB solution (40 mg/mL DHB in 50% methanol), which was used as the matrix solution, was sprayed over the tissue surface using a TM-Sprayer (HTX Technologies; Carrboro, NC, USA). The TM-Sprayer was also used to deliver a constant flow of heated sheath gas (N2, set at 10 psi) simultaneously with the matrix solution spray. The temperature of the sheath gas was maintained at 75 °C. The solvent pump system used was a Smartline P1000 (Knauer, Berlin, Germany) operated at a flow rate of 0.3 mL/min. The TM-Sprayer software was used for the system operations. Tissue sections were spray coated with the matrix solution so that extraction and co-crystallization could be performed simultaneously. MALDI-IMS analyses were performed using a MALDI-Fourier transform ion cyclotron resonance type instrument (Solarix XR; Bruker Daltonics) equipped with a Bruker Smartbeam-II™ Laser. The laser was set to the small spot size with 50% laser power. Mass spectra ranging from mass-to-charge ratio (m/z) 400–1200 were collected. The laser scan pitch was set to 50 μm.

### Statistical analysis

Statistical analyses were performed using SPSS version 25 statistical software (Chicago, IL, USA). The Student’s t-test and Spearman’s correlation test were used to compare mean values and correlations between two groups. A *P* value < 0.05 was considered significant.

### Abbreviation for glycerophospholipids

The structures of glycerophospholipid species are described in accordance with the nomenclature of LIPID MAPS (https://www.lipidmaps.org/).

## Supplementary Information

Supplementary Legends.

Supplementary Information 1.

Supplementary Information 2.

Supplementary Information 3.

Supplementary Information 4.

Supplementary Information 5.
