# Supplementary material for: Hypertrophy of the ligamentum flavum in lumbar spinal canal stenosis is associated with abnormal accumulation of specific lipids
Source: Sci Rep. 2021 Dec 6;11:23515. doi: 10.1038/s41598-021-02818-7 (PMC8648848; doi:10.1038/s41598-021-02818-7)
Supplement: Supplementary file 1 — Supplementary Legends. [file 41598_2021_2818_MOESM1_ESM.docx]

**Supplemental figure 1**

A vertebral arch removed from a patient during surgery in this study.

For analysis, only the ligament tissue surrounded by the dotted white line was used in this study.

**Supplemental figure 2**

Representative chromatogram for non-hypertrophied ligamentum flavum (NHLF) group and hypertrophied ligamentum flavum (HLF).

**Supplemental figure 3**

Dot plot showing the total amount of specific lipids of both non-hypertrophied ligamentum flavum (NHLF) group and hypertrophied ligamentum flavum (HLF).

(A) PCs, OAHFAs, and TGs, (B) PCs and OAHFAs, (C) PCs and TGs, (D) TGs and OAHFAs.

**Supplemental figure 4**

Negative ion mode MS/MS spectrum of OAHFA(16:0_24:0)-H; [M-H]^-^ (m/z 649.6) with retention time (RT) = 43.90 min from hypertrophied ligamentum flavum (HLF). The [M-H]^-^ accompanied by fragments consisted with deprotonated palmitic acid; [FA-H]^-^.
