# Supplementary figures and images for: Hypertrophy of the ligamentum flavum in lumbar spinal canal stenosis is associated with abnormal accumulation of specific lipids
Source: Sci Rep. 2021 Dec 6;11:23515. doi: 10.1038/s41598-021-02818-7 (PMC8648848; doi:10.1038/s41598-021-02818-7)

## Supplemental figure 1

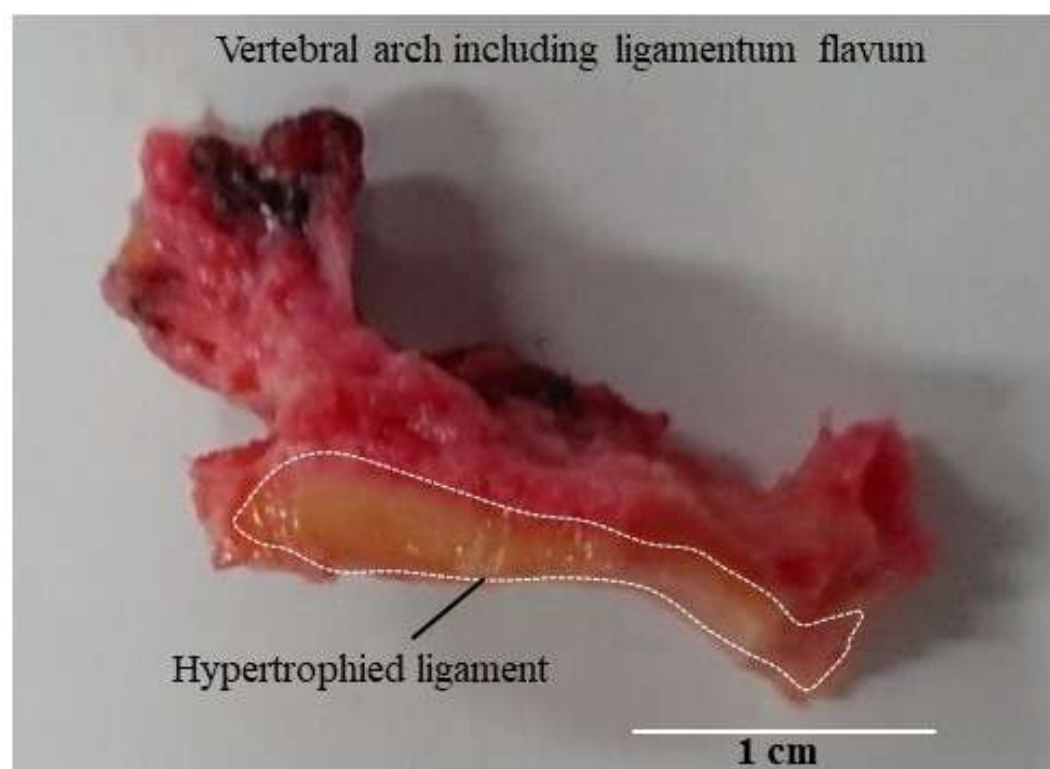

Supplement: Supplementary file 2 — Supplementary Information 1. [file 41598_2021_2818_MOESM2_ESM.pdf]

## Supplemental figure 2

RT: 0.00 - 60.01

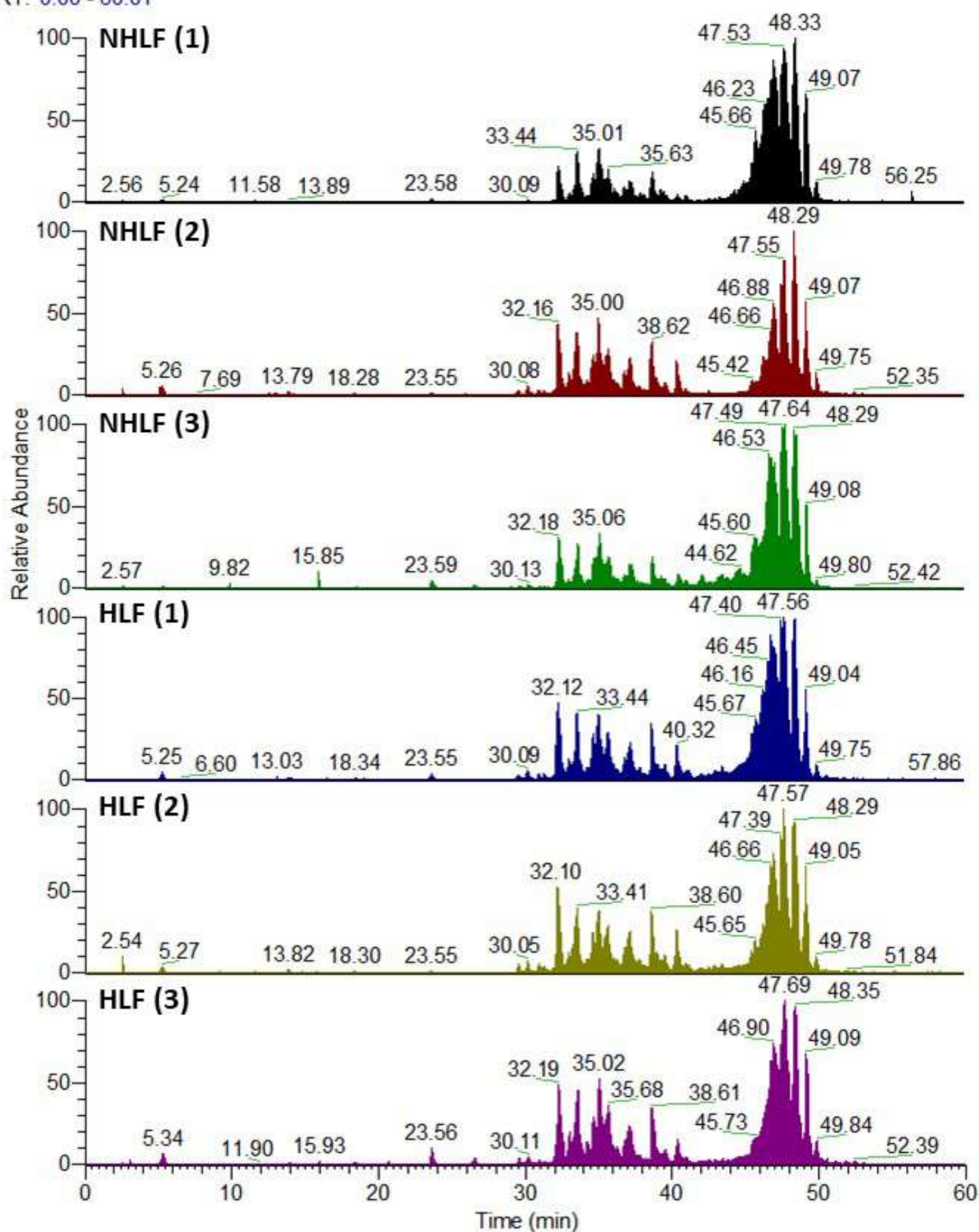

Supplement: Supplementary file 3 — Supplementary Information 2. [file 41598_2021_2818_MOESM3_ESM.pdf]

### Supplemental figure 3

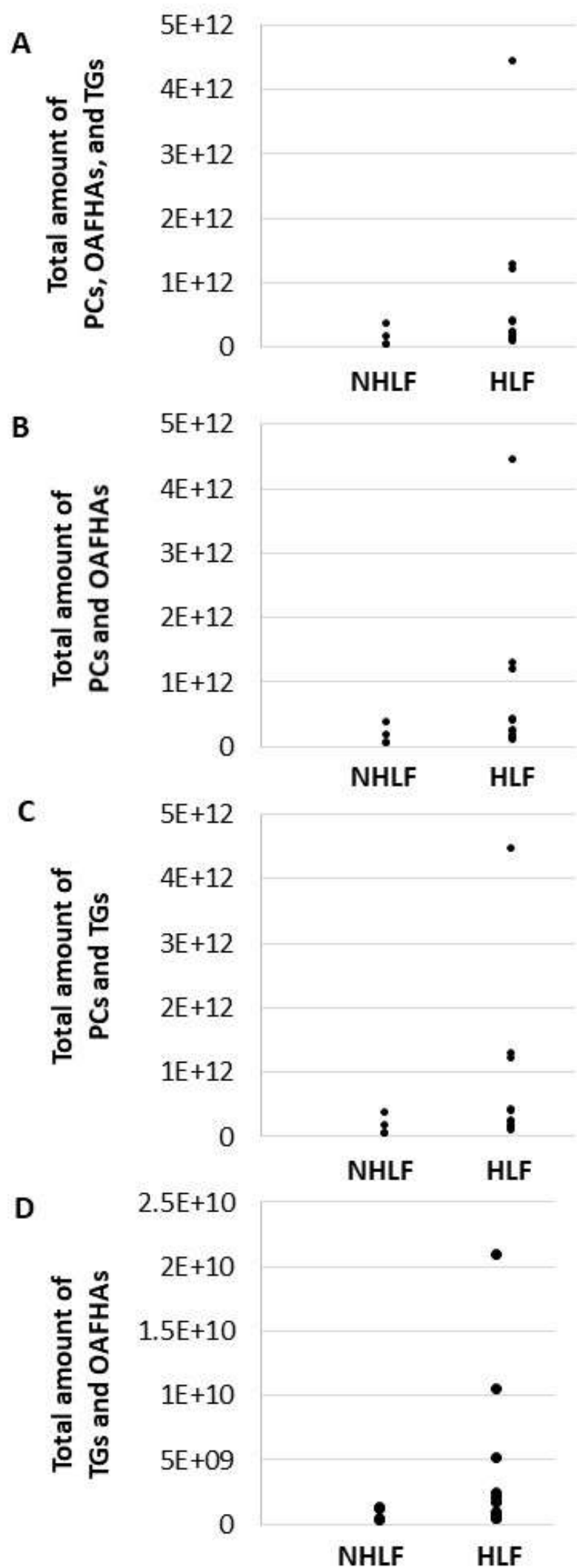

Supplement: Supplementary file 4 — Supplementary Information 3. [file 41598_2021_2818_MOESM4_ESM.pdf]

Supplemental figure 4

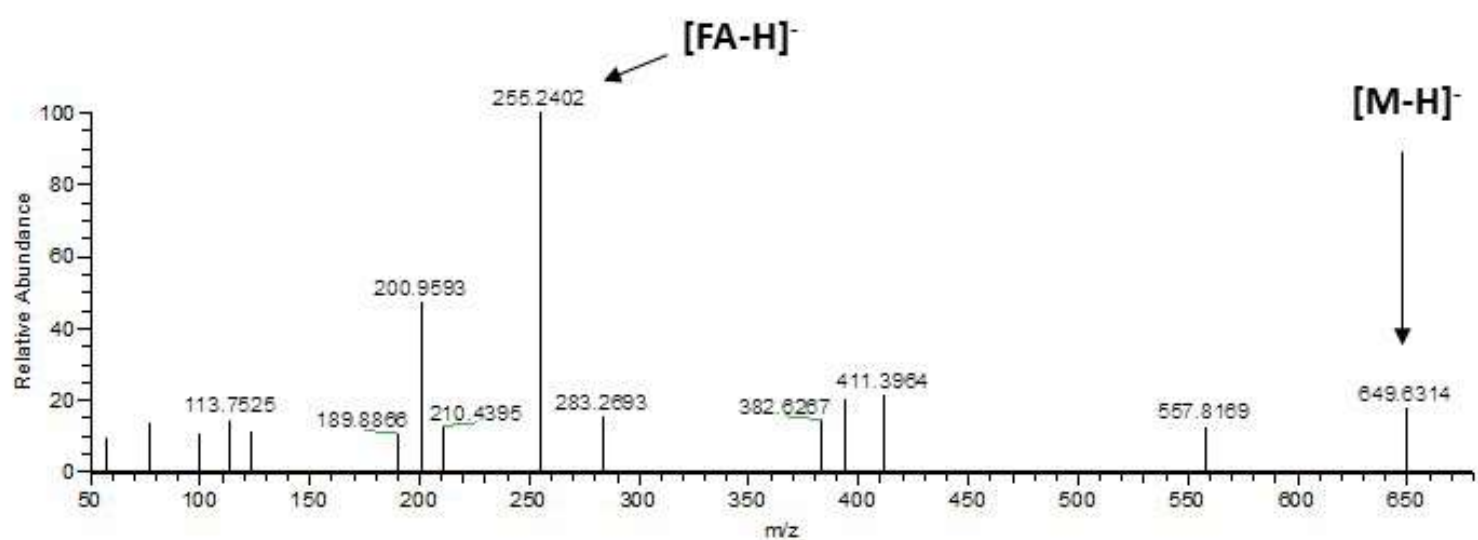

Supplement: Supplementary file 5 — Supplementary Information 4. [file 41598_2021_2818_MOESM5_ESM.pdf]
