# Supplementary material for: Hypertrophy of the ligamentum flavum in lumbar spinal canal stenosis is associated with abnormal accumulation of specific lipids
Source: Sci Rep. 2021 Dec 6;11:23515. doi: 10.1038/s41598-021-02818-7 (PMC8648848; doi:10.1038/s41598-021-02818-7)
Supplement: Supplementary file 6 — Supplementary Information 5. [file 41598_2021_2818_MOESM6_ESM.docx]

HLF: hypertrophied ligamentum flavum, NHLF: non-hypertrophied ligamentum flavum, FC: fold change

PC: phosphatidylcholine, Cer: ceramide, OAFHA: O-acyl-ω-hydroxy fatty acids, TG: triglyceride, PI: phosphatidylinositol, ZyE: zymosteryl, LPC: lysophosphatidylcholine, SM: sphingomyelin, ChE: cholesteryl ester, PG: phosphatidylgrycerol.
